# Elevated Atmospheric Carbon Dioxide Concentrations Amplify *Alternaria alternata* Sporulation and Total Antigen Production

**DOI:** 10.1289/ehp.0901867

**Published:** 2010-05-13

**Authors:** Julie Wolf, Nichole R. O’Neill, Christine A. Rogers, Michael L. Muilenberg, Lewis H. Ziska

**Affiliations:** 1 Department of Environmental Science and Technology, University of Maryland, College Park, Maryland, USA; 2 U.S. Department of Agriculture–Agricultural Research Service, Beltsville Agricultural Research Center, Beltsville, Maryland, USA; 3 Department of Public Health, University of Massachusetts, Amherst, Massachusetts, USA

**Keywords:** allergic rhinitis, Alternaria alternata, asthma, Cladosporium phlei, elevated atmospheric carbon dioxide (CO_2_), fungal antigenic protein, fungal sporulation, global climate change, plant carbon-to-nitrogen ratio (C:N), timothy grass (Phleum pratense)

## Abstract

**Background:**

Although the effect of elevated carbon dioxide (CO_2_) concentration on pollen production has been established in some plant species, impacts on fungal sporulation and antigen production have not been elucidated.

**Objective:**

Our purpose was to examine the effects of rising atmospheric CO_2_ concentrations on the quantity and quality of fungal spores produced on timothy (*Phleum pratense*) leaves.

**Methods:**

Timothy plants were grown at four CO_2_ concentrations (300, 400, 500, and 600 μmol/mol). Leaves were used as growth substrate for *Alternaria alternata* and *Cladosporium phlei*. The spore abundance produced by both fungi, as well as the size (microscopy) and antigenic protein content (ELISA) of *A. alternata,* were quantified.

**Results:**

Leaf carbon-to-nitrogen ratio was greater at 500 and 600 μmol/mol, and leaf biomass was greater at 600 μmol/mol than at the lower CO_2_ concentrations. Leaf carbon-to-nitrogen ratio was positively correlated with *A. alternata* spore production per gram of leaf but negatively correlated with antigenic protein content per spore. At 500 and 600 μmol/mol CO_2_ concentrations, *A. alternata* produced nearly three times the number of spores and more than twice the total antigenic protein per plant than at lower concentrations. *C. phlei* spore production was positively correlated with leaf carbon-to-nitrogen ratio, but overall spore production was much lower than in *A. alternata*, and total per-plant production did not vary among CO_2_ concentrations.

**Conclusions:**

Elevated CO_2_ concentrations often increase plant leaf biomass and carbon-to-nitrogen ratio. Here we demonstrate for the first time that these leaf changes are associated with increased spore production by *A. alternata*, a ubiquitous allergenic fungus. This response may contribute to the increasing prevalence of allergies and asthma.

Anthropogenic increases in global atmospheric carbon dioxide (CO_2_) concentration have been shown to stimulate earlier and greater production of allergenic pollen (LaDeau and Clark 2006; Rogers et al. 2006; Ziska et al. 2003), as have warming temperatures (Wan et al. 2002; Yli-Panula et al. 2009). The effects of anthropogenic climate change on the production of airborne fungal spores are not as well documented, but the implications for allergic disease are no less important. As with pollen, exposure to fungal spores is associated with allergy and asthma symptoms [Institute of Medicine (IOM) 2000, 2004; Salo et al. 2006], although the specifics of the relationship are not completely understood (Portnoy et al. 2008). Among patients with asthma from six regions of the world, 11.9%, on average, were sensitized to *Alternaria alternata*, with the proportion as high as 28.2% in Portland, Oregon; in addition, sensitivity to *A. alternata* was more prevalent among patients with more severe asthma (Zureik et al. 2002). Allergenic fungal spores may be increasingly abundant in some areas of the globe as well. In Derby, United Kingdom, mean seasonal airborne spore concentrations of the genus *Alternaria* have increased over the years 1970–1998, as have the number of days with spore counts > 50 per cubic meter of air, and the start of the *Alternaria* spore season has advanced from the end to the beginning of June (Corden and Millington 2001).

Rising atmospheric CO_2_ concentration is well documented [Intergovernmental Panel on Climate Change (IPCC) 2007]. Large increases in atmospheric CO_2_ concentration (e.g., doubled or greater increases in concentration) have been shown to alter both plant biomass and chemistry in many plant species (Ainsworth and Long 2005; Taub et al. 2008). The responses of individual plant species to large increases in atmospheric CO_2_ concentration are variable but often include greater total biomass production (de Graaf et al. 2006) and greater carbon-to-nitrogen ratios (C:N) of plant tissues (Taub and Wang 2008). Primary consumers, including allergenic fungi that grow on living or dead plant materials, are therefore likely to encounter changes in substrate quality or quantity as global atmospheric CO_2_ concentration increases (Reich et al. 2006).

The responses of plant–fungal interactions to increased atmospheric CO_2_ concentration may be more complex and difficult to elucidate than the observed changes in pollen production and allergenicity. Fungi play several different roles in plant biology, acting as plant pathogens, saprobes (decomposers of dead plant material), or plant symbionts, with neutral, beneficial, or negative impacts on living plant hosts. There may be a number of indirect effects on fungal growth and reproduction as a result of plant changes under increased atmospheric CO_2_ concentration as well as feedback and interactions among plants, fungi, and abiotic (e.g., environmental, nonorganismal) factors (Rillig 2007; Stiling and Cornelissen 2007).

In a field experiment using open-top chambers to grow individual poplar trees in ambient or doubled atmospheric CO_2_ concentration, Klironomos et al. (1997) found that air and leaf litter in chambers with doubled CO_2_ concentration contained significantly more fungal spores than those in ambient chambers. An indirect effect of doubled atmospheric CO_2_ concentration on sporulation, mediated by changes in plant chemistry, was suggested by their findings; however, a direct effect of atmospheric CO_2_ concentration or of related changes such as soil moisture and relative humidity could not be ruled out.

To directly assess the quantitative and qualitative impacts of rising atmospheric CO_2_ concentration on allergenic fungi, we grew the perennial C_3_ monocot timothy grass (*Phleum pratense*), a common fodder used for hay, at four levels of atmospheric CO_2_ concentration approximating preindustrial, current, and potential/projected future concentrations (300, 400, 500, and 600 μmol/mol, respectively). We used leaves from these plants to separately grow two fungal species. The first species, *A. alternata*, is a ubiquitous, facultatively plant-pathogenic or saprobic species known to produce allergenic airborne conidial spores (Sanchez and Bush 2001). The second species, *Cladosporium phlei* (syn. *Heterosporium phlei*), is a specific pathogen of timothy that has not been specifically shown to produce allergenic spores; other species within the genus *Cladosporium* are known to cause allergies in humans (Kurup 2003; Poll et al. 2009). We also examined the sporulation of *A. alternata* on clippings from field-grown grasses to see if responses were similar on other grass species. Our objective was to quantify changes in sporulation on plant leaves grown at varying atmospheric CO_2_ concentrations and elucidate the mechanism that drives such changes.

## Methods

### General approach

We used two growth chambers to provide atmospheric CO_2_ concentration at 300, 400, 500, or 600 μmol/mol to experimental timothy plants for 60 days of growth. These approximate atmospheric CO_2_ concentrations at the beginning of the 19th century, current ambient levels, and those projected by the years 2025 and 2040, respectively (IPCC 2007). During repeated runs over time, each of the two growth chambers was used for each level of CO_2_ concentration twice. During each run, each chamber contained 10 timothy plants. After 60 days of growth, leaf subsamples from each plant were used to grow *A. alternata* and *C. phlei* separately for 7 days inside loosely capped media bottles in an incubator. Media bottles were then volumetrically flooded with water, shaken, and subsampled for spore counts and, for *A. alternata* only, for antigenic protein extraction and quantification via ELISA using polyclonal antibodies.

### Growth, harvest, and C:N quantification of timothy grass

The study was conducted using two controlled environment chambers (EGC Corp., Chagrin Falls, OH) at Beltsville, Maryland, with a given chamber set at one of four atmospheric CO_2_ concentration set points (300, 400, 500, and 600 μmol/mol) for 24 hr/day. The atmospheric CO_2_ concentration of the air within each chamber was controlled by adding either CO_2_ or CO_2_-free air to maintain the set concentration. Actual average 24-hr atmospheric CO_2_ concentration values (± SD) were 315 ± 19, 395 ± 11, 491 ± 10, and 589 ± 12 μol/mol. Injection of CO_2_ and CO_2_-free air was controlled by a TC-2 controller using input from an absolute infrared gas analyzer (WMA-2, PP Systems, Haverhill, MA). Chamber temperatures were altered diurnally (in steps) from an overnight low of 20°C to a maximum afternoon value of 30°C. Photosynthetically active radiation (PAR) was altered concurrently with temperature, with the highest PAR value (900–1,000 μmol/m^2^ per sec) occurring during the afternoon (1,200–1,500). The duration of daily PAR was 14 hr, which was supplied by a mixture of high-pressure sodium and metal halide lamps.

Seeds of timothy grass (*P. pratense*, var. Climax) were obtained from Pawnee Buttes Seed (Greeley, CO). We sowed three seeds in each 4-inch pot used and thinned to one seedling per pot 6–8 days after emergence. Pots were watered to the drip point daily with a complete nutrient solution containing 14.5 mmol/L nitrogen. Ten pots were grown in each of the two chambers during each run. In eight runs over time, each level of CO_2_ concentration was run a total of four times, twice in each chamber. Plants from the third run were stunted and grew poorly for unknown reasons; this run was omitted, resulting in three runs for CO_2_ concentration levels of 300 and 500 and four runs for CO_2_ concentration levels of 400 and 600.

Plants were harvested 60 days after sowing. All leaves were separated from stems above the collar and weighed. Ten leaves from each plant were used for determination of total area, mass, and nitrogen and carbon content, which was determined using a PerkinElmer 2400 Series II CHNS/O analyzer (PerkinElmer, Waltham, MA). Leaf subsamples of approximately 2 g fresh weight were removed from each plant for fungal growth, refrigerated, and processed within 3 hr of harvest.

All solutions, suspensions, and rinses were made using sterilized, deionized, distilled water (ddw) with Tween 80 (Fisher Scientific, Fairlawn, NJ) added at the rate of 5 drops/L (ddw+T). Leaves were surface-sterilized by immersion and agitation in 1 L of 0.3% sodium hypochlorite solution for 3 min, followed by three successive 1-min rinses. All solutions and rinses were changed after leaves from all plants in a chamber were immersed to avoid transfer of any leaf material among treatments. Surface-sterilized leaves were oven-dried at 68°C for 48 hr.

### Inoculum preparation, leaf inoculation, and fungal growth

The fungal isolates grown were *A. alternata* (Fries) Keissler, isolate EGS 34-016, obtained from the collection of E. Simmons, and *C. phlei* (C.T. Greg.) G.A. de Vries, isolate CBS 306.5, obtained from the Fungal Biodiversity Centre of the Centraalbureau voor Schimmelcultures (Utrecht, the Netherlands). Isolates were cultured on standard petri plates with half-strength V8 juice agar (100 mL V8 juice; Campbell’s Soup Company, Camden, NJ), 900 mL ddw, 2 g CaCO_3_, 15 g agar powder, autoclaved for 20 min at 121°C).

Fungal inocula were prepared immediately before inoculating leaves and prepared the same way for both fungal species. Two Petri plates with conidia covering most of the agar surface were selected. Conidia were removed from these plates by pouring small aliquots of ddw+T onto the surface of the agar in each plate, gently scraping the agar surface with a ceramic scraper, pouring the water with removed conidia through four layers of sterilized cheesecloth (Fisher Scientific) into a sterilized 1-L beaker, and repeating three more times. The resulting spore suspensions were vortexed (Vortex Genie2, Daigger, Vernon Hills, IL) at maximum speed for 30 sec to separate clusters of spores and subtending hyphae. The total volume was brought up to 1 L with additional ddw+T. Inoculum strength was standardized visually: A 10-mL subsample was removed with a glass wide-mouth pipette, filtered with a 47-mm diameter, 0.45-μm pore size, gridded mixed-cellulose filter (Fisher Scientific) in a glass vacuum filtration apparatus, and examined at 5× magnification under a dissecting microscope (SMZ 1500; Nikon Precision Inc., Belmont, CA). If spores were more than one layer deep on the filter, ddw+T was added to the inoculums suspension in 200-mL increments until subsamples resulted in filters densely covered with a single layer of spores. Five hundred milliliters of the final inoculum suspension was placed into two sterilized 1-L beakers; each beaker was used to inoculate leaves from a single chamber.

Weighed leaf subsamples (0.5 ± 0.075 g dry weight) from each single plant were inoculated separately by immersion and agitation in the inoculum suspension for 1 min. Inoculated leaves were placed in a sterilized media bottle (250-mL size used for *A. alternata*, 500-mL size for *C. phlei*) (VWR, West Chester, PA) with caps placed loosely, then incubated for 1 week in an environmental controller (Percival, Perry, IA) set at 21°C with a 12-hr light/dark cycle and ambient CO_2_ concentration.

### Spore harvest and A. alternata antigen extraction

After 1 week, 200 mL sterilized ddw+T was added to each media bottle. Bottles were vortexed at maximum speed for 30 sec to dislodge spores from leaves and subtending hyphae. For *A. alternata* only, antigen was then extracted: media bottles were left at room temperature for 2 hr; bottles were then inverted several times by hand. With a sterile syringe, 20 mL water with suspended spores was removed from each bottle and passed through a syringe filter (0.22-μm pore size, 33-mm diameter, polyethersulfone; Millipore, Billerica, MA). The resulting spore-free extract was frozen inside a 50-mL plastic centrifuge tube (Corning Inc., Corning, NY) and lyophilized on a Freezone 4.5 freeze dry system (Labconco, Kansas City, MO). For both fungal species, media bottles were then inverted several times by hand; 10-mL subsamples were removed using glass wide-mouth pipettes, placed on filters, and examined as described above. If spore density on the filter was too sparse (or too crowded) for accurate counting, a larger (or smaller) subsample was obtained, a new filter was made, and spore density was rechecked. The final volume used for each subsample ranged from 1 to 100 mL and was recorded for extrapolation of the total number of spores produced per gram of dried leaf tissue.

### Spore quantification

Filters were rewetted from below with several drops of sterilized ddw and placed on a compound light microscope stage (Axioplan2 Imaging Microscope; Carl Zeiss Microimaging Inc., Thornwood, NY). Eight 300 × 400 μm fields of view were digitally photographed (Axiocam digital camera and Axiovision imaging software; Carl Zeiss Microimaging, Inc.) at random positions over one half of each wetted filter. Spores within each of the eight fields were counted manually from the photographs; all whole, uncollapsed spores were counted regardless of size. For *A. alternata* only, the first 10 spores laying flat on the filter were also measured for length and width.

### Antigen quantification

Lyophilized extracts were reconstituted in 3 mL ddw and assayed for *A. alternata* antigen using a competitive inhibition ELISA described by Salo et al. (2005). The assay kit consisted of a rabbit polyclonal antibody (lot ZA4-4; Greer Laboratories, Lenoir, NC) produced using whole mycelial extracts of *A. alternata* (lot XPM1-X10); the *A. alternata* mycelial extract was also used as the standard in the assay. This assay has been shown to detect multiple *A. alternata* antigens including the allergen Alt a 1 (Portnoy et al. 1993; Salo et al. 2006). Briefly, the standard *A. alternata* extract was bound to plastic microtiter plate wells, excess was washed away, and unbound sites were blocked with 1% bovine serum albumin solution. Dilution series of the sample extracts were pipetted into the wells and followed immediately by the rabbit anti-*A. alternata* antibody. After incubation, we aspirated solutions, washed the wells, and added a peroxidase-labeled goat anti-rabbit antibody solution (Sigma-Aldrich, St. Louis, MO). Finally, we added substrate and measured the color change at 405 nm using a microtiter plate reader (Molecular Devices, Sunnyvale, CA). We compared the resulting reaction rates against a dilution series of standard *A. alternata* antigen to determine antigen concentration. Results are reported as micrograms *A. alternata* antigen per spore or per plant.

### Data analysis

All statistical analyses were conducted using R version 2.8.1 (R Development Core Team 2008). The R package “lme4” (Bates et al. 2008) was used to fit and evaluate generalized linear mixed models of the following effects: atmospheric CO_2_ concentration level (fixed at 300, 400, 500, or 600 μmol/mol), run (random effect of seven runs over time), and chamber (random effect of the two growth chambers used). All fungal responses were also modeled with the covariate of leaf C:N of each plant. Factors were sequentially removed from models and model fit was evaluated using Akaike’s information criterion; for each response, the simplest statistical model with good fit was used. We used the R package “languageR” (Baayen 2008) to estimate *p*-values for the fixed effect of atmospheric CO_2_ concentration level in each model using Markov Chain Monte Carlo resampling and to generate highest posterior density confidence intervals (CIs) for means at each atmospheric CO_2_ concentration level. We considered *p*-values ≤ 0.05 to be statistically significant. The responses evaluated were the following: *a*) *A. alternata* spore counts (natural log transformed to meet statistical model assumptions that variance does not increase with group mean); *b*) mean length and width of *A. alternata* spores; *c*) quantity of antigenic protein produced (square-root transformed to linearize relationships with model factors and to meet statistical model variance assumptions); and *d*) *C. phlei* spore counts (natural log transformed, as above).

The random effect of chamber was neither significant nor needed to improve model fit for any responses and is not discussed further. The random effect of run was very important for modeling all plant and fungal responses; model-adjusted means, which account for variation due to run, are presented for responses at individual levels of CO_2_ concentration. When the fixed effect of CO_2_ concentration was determined to be statistically significant, the R package “multcomp” (Hothorn et al. 2008) was used to run pairwise Tukey’s comparisons of CO_2_ concentration level means. The contribution of each factor toward explaining response variance was calculated as described by Kramer (2005).

### Supplemental experiment with field- collected plant material

**Figure 1:**
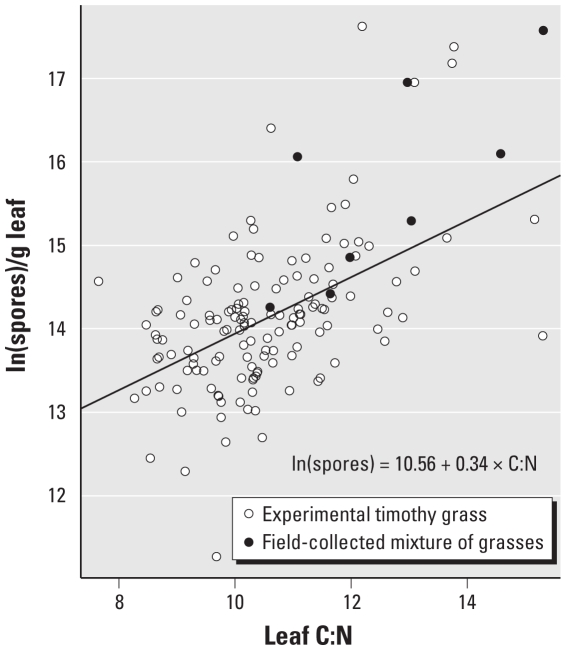
Effect of increasing leaf C:N on the natural log of *A. alternata* spores produced per gram of *P. pratense* leaf tissue. Field-collected mixtures are *L. perenne* and *P. annua*.

Nine samples of plant material were collected during routine mowing from three different sites at each of three local golf courses. A mixture of perennial ryegrass (*Lolium perenne* L.) and annual poa (*Poa annua*), both cool season grasses with C_3_ photosynthetic metabolism, is grown at all sites, but fertilizer application rates vary. The samples were collected on 8 January 2008 and refrigerated within 3 hr of mowing. Leaves were treated and used as growth substrate for the two fungal species as described above. Resulting C:N and spore counts were not statistically analyzed because of small sample size but are shown for visual comparison (Figure 1).

## Results

### Plant responses to atmospheric CO_2_ concentration levels

Leaf C:N was significantly higher in plants grown at 500 and 600 μmol/mol atmospheric CO_2_ concentrations than at the two lower concentrations (*p* = 0.017) (Table 1). Leaf dry weight per plant was significantly greater at 600 μmol/mol atmospheric CO_2_ concentration than at the three lower concentrations (*p* < 0.001) (Table 1). Specific leaf area was not significantly affected by atmospheric CO_2_ concentration (not shown). Plant responses were variable among individual plants and runs (Table 2). Among individual plants from all treatment levels, leaf C:N ranged from a minimum of 7.7 to a maximum of 16.2, which corresponds to total leaf nitrogen contents of 5.6% and 2.8%.

### General fungal growth

Inoculations of all experimental plants were successful for both fungal species. No spores other than those of the inoculated fungal species, and no other contaminants, were observed in any measurements of fungal growth. All antigen extracts were above the lower limit of antigen detection (LLOD) of the inhibition assay; the LLOD was always < 0.050 μg/mL and was < 0.011 μg/mL for most samples. Fungal responses also varied across individual plants and runs (Table 2).

### Fungal sporulation on leaves grown at four atmospheric CO_2_ concentration levels

**Figure 2:**
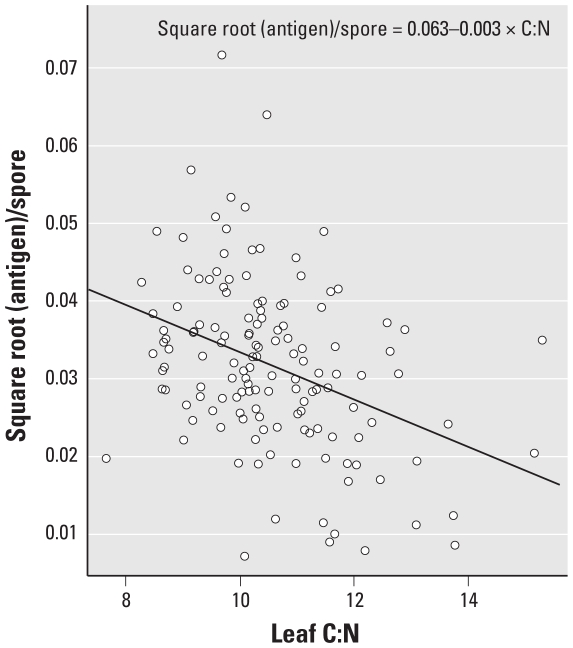
Effect of increasing *P. pratense* leaf C:N on the *A. alternata* spore antigen content (square-root transformed).

The log-transformed counts of *A. alternata* spores produced per gram leaf were positively correlated with leaf C:N (*p* < 0.001; adjusted *R*^2^ = 0.25; Figure 1), with an order-of-magnitude increase across the range of leaf C:N of experimental plants. *A. alternata* sporulation on field-collected grass leaves (supplemental experiment) shows a similar relationship (Figure 1). Mean length and width of *A. alternata* spores did not change significantly with leaf C:N (not shown). In contrast to the positive association with spore numbers, the quantity of *A. alternata* antigen per spore (square-root transformed) decreased as leaf C:N increased (*p* < 0.001; adjusted *R*^2^ = 0.34; Figure 2). Despite the decreased antigen content per spore at higher C:N, the quantity of *A. alternata* spores as well as the total antigen produced on a per-plant basis were positively associated with atmospheric CO_2_ concentration levels beyond the variation attributed to leaf C:N, with nearly three times the number of spores and twice the amount of antigen produced on plants grown at 500 and 600 μmol/mol atmospheric CO_2_ concentrations (*p* < 0.001; Table 1) than at the lower two concentrations.

**Figure 3:**
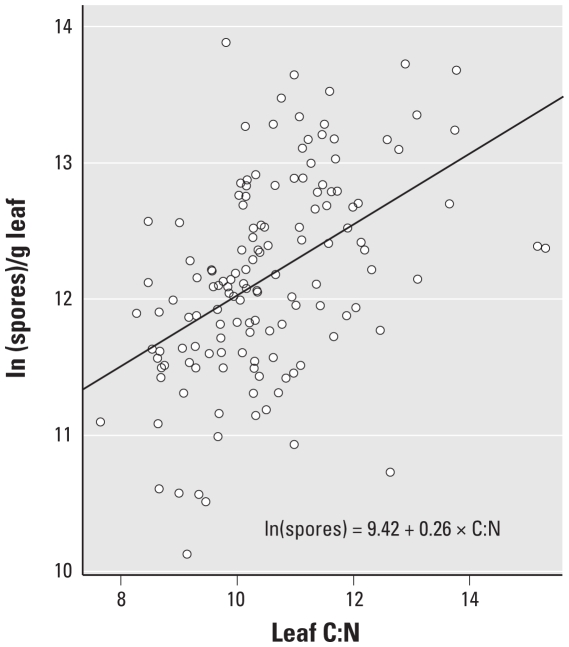
Effect of increasing leaf C:N on the natural log of *C. phlei* spores produced per gram of *P. pratense* leaf tissue.

The log-transformed counts of *C. phlei* spores produced per gram leaf also increased with C:N (*p* < 0.001; adjusted *R*^2^ = 0.22; Figure 3). Spores of *C. phlei* were produced in much lower numbers than *A. alternata* spores, although the slopes of the relationships with leaf C:N were not significantly different between the two fungi (*T* = 1.21; *p* > 0.20). In contrast with *A. alternata* spores, however, the mean number of *C. phlei* spores produced on a per-plant basis was not significantly different among plants grown at different CO_2_ concentration levels (*p* = 0.78; Table 1).

## Discussion

Climate change and urbanization are expected to increase the prevalence of asthma and allergies (Schmier and Ebi 2009). Concomitant rises in temperature, atmospheric CO_2_ concentration, and pollen abundances over recent decades have been suggested as causes of increasingly prevalent and severe asthma and allergy symptoms observed over the same time period (Beggs and Bambrick 2005; Shea et al. 2008). Our findings indicate that, as with pollen production, the sporulation of allergenic fungi is likely to be amplified as atmospheric CO_2_ concentration increases and therefore is also likely to contribute to increasing prevalence and severity of asthma and allergies.

We found significant positive relationships between leaf C:N and *A. alternata* and *C. phlei* spore production per gram leaf, although only *A. alternata* sporulation was associated with CO_2_ concentration level after accounting for changes in leaf C:N. These relationships suggest that considerable increases in sporulation of both species will occur, and may already be occurring, if rising atmospheric CO_2_ concentration increases plant C:N in the field. The response of *C. phlei*, however, may not be as strong as that of *A. alternata*. More study of this species is needed.

Although C:N was correlated with increased sporulation, a large proportion of the variability in spore production remains unexplained. Some of this variability in sporulation may result from inherent variation in growth among individual plants, which can be of a magnitude large enough to obscure responses to elevated atmospheric CO_2_ concentration treatments (Poorter and Navas 2003). Other aspects of environmental variability, which deserve additional scrutiny, may also contribute to variation in spore numbers and quality. Temperature was kept constant during fungal growth in this experiment, but relative humidity was not controlled. Additional plant attributes such as leaf tissue concentrations of mineral elements (other than nitrogen), stomatal density, and other leaf surface properties, and the chemistry of leaf carbon compounds were not measured. All of these factors may contribute to the variation in sporulation, and all are also likely to be altered by anthropogenic climate change (Denman et al. 2007). For example, plant transpiration is likely to decrease under elevated atmospheric CO_2_ concentration, which often leads to increased moisture in soils and overlying litter (Johnson et al. 2003); warming temperatures and altered precipitation patterns will further impact moisture in soil and plant litter layers. Global climate change factors encompass many direct and indirect effects on plants and fungi, as well as complex interactions and feedbacks. The net effects of global changes are difficult to capture with a single study. Long-term (multidecadal) observational studies are needed to distinguish the impact of multiple abiotic changes on allergenic spore production *in toto*.

Our results corroborate the findings of Klironomos et al. (1997), who found large increases (~ 100–150%) in *Alternaria* spore abundance in chambers with poplar trees growing in elevated atmospheric CO_2_ concentration. Our results also suggest the potential ubiquity, across plant species, of increased spore production as a function of increasing atmospheric CO_2_ concentration. Klironomos et al. (1997) also found a large increase in the abundance of spores from the genus *Cladosporium* at elevated atmospheric CO_2_ concentration (~ 225–350% increases), which was not apparent in our study. A strong response to atmospheric CO_2_ concentration may relate to a saprobic (growing on dead plant tissues) lifestyle. *A. alternata* is facultatively pathogenic or saprobic. Although *C. phlei* can grow on dead plant tissues, as it did in this experiment, it is described primarily as a pathogen of living timothy grass. Other species within *Cladosporium,* however, are saprobic (Zalar et al. 2007). Specializations that allow fungal species to grow on specific host plants, such as unique metabolic pathways, may cause these fungi to be limited more by other plant characteristics than by carbon content alone.

Although only a few studies have examined fungal sporulation on plants grown at elevated atmospheric CO_2_ concentration, there has been extensive work examining sporulation on artificial growth media and on plants with different levels of nitrogen content. In some pathogenic fungal species, sporulation on living leaves is greatest at medium C:N (Walters and Bingham 2007), but other species sporulate maximally at high C:N. The C:N that promotes the most sporulation varies among fungal species and even strains (Elson et al. 1998; Gao et al. 2007). These varying trends among fungal taxa may reflect different reproductive strategies, specializations, and functional tradeoffs needed to thrive in different environmental and host conditions (Pariaud et al. 2009). There is experimental evidence that while the greatest numbers of spores may be produced at high C:N in some fungal taxa, spore protein content, germination success, and host-plant infectivity are greater when more nitrogen is available (Jackson and Schisler 1992; Yu et al. 1998). In our study, the decreasing content of antigenic protein in *A. alternata* spores grown at higher C:N suggests increasing nitrogen limitation of the fungus. It is not yet clear if this nitrogen limitation is associated with compromised germination, growth, or allergenicity of individual *A. alternata* spores.

Meta-analyses of plant responses to elevated atmospheric CO_2_ concentration reveal clear trends despite variation among plant species and experimental conditions. The mean increase in C:N across many studies of grass species grown at doubled atmospheric CO_2_ concentration was 24.4% (Taub and Wang 2008). The responses of plants to less-than-doubled increases in atmospheric CO_2_ concentration have not been well studied, so expectations for C:N of plant tissues grown in real-world, gradually increasing atmospheric CO_2_ concentration cannot be predicted with certainty. However, an observational study of herbarium leaf specimens found that leaf nitrogen content decreased by a mean of 17% across samples from the early, mid, and late 20th century for 11 C_3_ plant species (Penuelas and Estiarte 1997) [recorded global levels of atmospheric CO_2_ concentration increased from < 320 in 1958 to 388 μmol/mol in July 2009 (National Oceanic and Atmospheric Administration 2009)]. The authors attribute the decreased leaf nitrogen content to the combination of direct and indirect (warming) effects of rising atmospheric CO_2_ concentration, and their findings are corroborated by other observational and experimental measurements (Denman et al. 2007; Gifford et al. 2000; Taub et al. 2008; Taub and Wang 2008). Thus, this evidence of increased plant C:N associated with rising atmospheric CO_2_ concentration and climatic change is consistent with increased sporulation of allergenic fungi, as observed in the current study, and has implications for increasing the prevalence and severity of allergy and asthma symptoms (Beggs and Bambrick 2005; D’Amato et al. 2005).

## Figures and Tables

**Table 1:** Adjusted means and 95% CIs by CO_2_ concentration level.

	CO_2_ concentration level (μmol/mol)			
	300	400	500	600
Leaf C:N	10.5 (9.7–11.1)	9.9 (9.3–10.6)	10.8 (10.1–11.5)	11.0 (10.3–11.7)
Total leaf dry weight (g)	9.7 (6.9–12.2)	9.6 (6.8–11.8)	9.4 (7.0–12.4)	13.6 (11.1–16.1)
*A. alternata* spores produced per plant (natural log transformed)	16.0 (15.6–16.4) [8,797,692]	15.7 (15.3–16.1) [6,388,435]	16.7 (16.2–17.1) [17,540,095]	17.0 (16.6–17.4) [23,207,823]
*A. alternata* antigen (micrograms) per plant (square-root transformed)	87.4 (67.0–105.3) [7,639]	93.3 (75.8–112.5) [8,705]	125.3 (106.0–144.5) [15,700]	140.6 (124.1–159.8) [19,768]
*C. phlei* spores produced per plant (natural log transformed)	14.6 (14.2–15.1) [2,191,288]	13.9 (13.5–14.3) [1,088,161]	14.5 (14.0–14.9) [1,982,759]	14.6 (14.2–15.0) [2,191,288]

a Model-adjusted means with highest posterior density 95% CIs are shown in parentheses. For natural log and square-root transformed responses, back-transformed means are shown in square brackets.

**Table 2:** Percent contribution of explanatory variables (factors) to variability of responses.

Response factor	Leaf C:N	Total leaf dry weight	*A. alternata* spores/plant	*C. phlei* spores/plant	*A. alternata* antigen/spore	*A. alternata* antigen/plant
[CO_2_]	6.0	7.7	9.0	4.4	0	15.7
Leaf C:N	NA	NA	9.7	11.4	8.4	4.6
Run	24.5	62.1	9.0	17.7	8.0	39.4
Residual	69.5	30.2	72.3	66.5	83.6	40.3
Total	100	100	100	100	100	100

NA, factor not analyzed. Percent contributions were calculated as described by Kramer (2005).
